# Burden of adult neurofibromatosis 1: development and validation of a burden assessment tool

**DOI:** 10.1186/s13023-019-1067-8

**Published:** 2019-05-03

**Authors:** Marie-Laure Armand, Charles Taieb, Aline Bourgeois, Mireille Bourlier, Mohammed Bennani, Christine Bodemer, Pierre Wolkenstein, along with the French national network on rare skin diseases (FIMARAD)

**Affiliations:** 1 Department of Dermatology, Hôpital Henri Mondor, Créteil, France; 2 FIMARAD, French rare diseases Healthcare Network: rare dermatologic disease, CHU Paris - Hôpital Necker-Enfants Malades, 149 rue de Sèvres, 75743 Paris, France; 3 Association Neurofibromatoses et Recklinghausen, patients association, Neuville-en-Ferrain, France; 4 Qualees, Paris, France; 5 MAGEC center of excellence for rare skin diseases, Necker-Enfants Malades University Hospital, Paris, France

**Keywords:** Neurofibromatosis type 1, NF1, Von Recklinghausen’s disease, Individual disease burden, Quality of life, Questionnaire

## Abstract

**Background:**

Neurofibromatosis Type 1 (NF1) is a common genetic neurocutaneous disease, with an autosomal dominant inheritance mode. Quality of life has been shown impaired in NF1, due to severe complications, cosmetic features, and uncertainty about the disorder.

**Methods:**

This study sought to develop a self-administered questionnaire in French to assess the burden of NF1 (BoN), then translate and linguistically and cross-culturally validate it into American English, standardized methodology applied, as outlined in the report.

**Results:**

Based on several discussions with NF1 patients, a 17-item conceptual questionnaire was first produced. Of the 91 NF1 adult patients who responded to the conceptual questionnaire, 65 (64.6% females) were accessible. Subsequent confirmatory analyses generated a 15-item questionnaire grouped into four domains, demonstrating internal consistency (Cronbach’s alpha: 091), discriminant validity, and high reliability. The BoN was likewise shown to significantly correlate with other validated questionnaires, such as Dermatology Life Quality Index, Perceived Stress Scale, and SF12 mental score, indicating good external validity.

**Conclusions:**

BoN is a specific tool for assessing the burden that NF1 generates on many practical aspects of the patient daily activities, beyond the notion of quality of life”. Given the increasing relevance that regulatory authorities attribute to patient-reported outcomes, the BoN questionnaire provides such supplementary information while accounting for the burden of NF1 patients in the broadest sense.

**Electronic supplementary material:**

The online version of this article (10.1186/s13023-019-1067-8) contains supplementary material, which is available to authorized users.

## Background

Neurofibromatosis Type 1 (NF1) is the most common autosomal dominant neurocutaneous disease, caused by mutations in the NF1 tumor suppressor gene, located in a region on chromosome 17ql1.2 [1]. This gene encodes a common protein, namely neurofibromin, which is an essential negative regulator of Ras cellular proliferation pathways [2, 3]. Disease prevalence is estimated at 1:2000–1:3500 individuals worldwide, yet with wide phenotypical variability [4–6]. The germline NF1 mutation rate proves ten-fold higher than that observed in other inherited disease genes [7].

Establishing the correct NF1 diagnosis may prove challenging [8]. NF1 patients are at increased risk of developing various tumors, and their life-expectancy is decreased by 10 years as compared to the general population [9]. With skin lesions as the most noticeable disease manifestation, NF1 may affect many organs, and psychiatric and psychological disorders are likewise common [10, 11]. Around 20% of patients display dysthymia and 7% depressed mood, with a heightened risk of suicide [12]. Anxiety and personality disorders are similarly observed, and cognitive disorders may last into adulthood, thereby impacting smooth integration into work [13]. Quality of life (QoL) was shown impaired in NF1 patients, especially in severe disease cases [14].

The concept of “burden” has taken a central role in evaluating care [15], and specifically in case of skin diseases [16]. In 2010, the World Health Organization first introduced the concept of “Global Disease Burden”, particularly useful for quantifying population health and determining action priorities [17]. The focus has meanwhile been switched to “individual disease burden”, a concept designed to assess disease “disability” in the broadest sense, including psychological, social, economic, and physical features. Such individual disease burden was already investigated in skin diseases like psoriasis [18], infantile hemangioma [19], hereditary ichthyosis [20], atopic dermatitis [21], and vitiligo [22].

Ferner et al. [23] have developed a disease-specific questionnaire to measure QOL in people with NF1 (INF1-QOL) that is suitable as an assessment tool in clinical practice and in clinical trials. The aim of our work is to assess the burden and the impact that the NF1 generates on many practical aspects of the patient daily activities in addition to the notion of quality of life.

As part of their research activities, the “reference centers for rare skin disorders” network has elaborated and validated a French questionnaire designed to assess the burden of NF1 on patients suffering from the condition, termed **B**urden **o**f **N**eurofibromatosis (BoN). This paper describes the different steps involved.

## Methods

The self-administered BoN questionnaire was developed using standard methodology comprising three phases, namely exploratory, development, and validation [24]. This questionnaire was developed by a multidisciplinary working group comprising experts in questionnaire design/development, such as healthcare professionals like physicians and psychologists, experts in NF1, such as social workers and dermatologists, as well as experts in QoL and patient-reported outcomes.

The questionnaire was conventionally created in a question and answer format. Response modalities were determined via consensus among the experts, and took the form of a 6-point Likert scale: “never” (0), “rarely” (1), “sometimes” (2), “often” (3), “very often” (4), and “constantly” (5). To limit missing data, we also considered a “not applicable” (0). Instructions in the preamble state that the questions relate to the previous 30 days. Subjects from the age of 15 could complete the questionnaire and had the option to indicate “not concerned” for non-applicable questions.

### Exploratory phase

The initial step involved several one-to-one interviews between the dermatologist, psychologist, social worker, and expert in patient-reported outcomes with patients suffering from NF1 to comprehensively collect the patients’ perceptions and complaints. By analyzing these interviews, the most relevant concepts were identified. A semi-structured questionnaire was then elaborated comprising specific themes in a question/answer format, including closed-ended questions with a choice of predetermined answers, as well as open-ended questions allowing unrestricted answers. The final choice of questions was made by the working group.

### Development phase

During this phase, the conceptual questionnaire was administered to a sample of subjects suffering from the disease. An exploratory factor analysis was conducted to highlight the underlying constructs, assigning each item to its respective domain, with orthogonal varimax rotation performed to verify whether the hypothetical constructs were interrelated.

To evaluate the questionnaire’s internal consistency and confirm its reliability, the item homogeneity in each dimension was tested using Cronbach’s alpha coefficient [25]. Higher order factor confirmatory analysis was performed to confirm that the dimensions created could be combined into one single score. PROC CALIS procedure, SAS 9.4, was employed. The criteria for the model’s goodness-of-fit were defined as a Bentler comparative fit index > 0.90, and Bentler-Bonett non-normed fit index > 0.90 [26], with root mean square error of approximation (RMSEA) close to 0.05 or at the very least less than 0.08.

### External validity

To assess the questionnaire’s external validity, all participants were asked to complete three previously validated self-administered questionnaires: the D*ermatology Life Quality Index* [DLQI] questionnaire,

The DLQI questionnaire is the first dermatology-specific instrument designed to assess the impact of skin diseases on patient QoL [27, 28]. Intended for adults and patients aged over 16 years, DLQI has proven a simple, practical, and validated questionnaire that grades QoL, the DLQI score being the sum of all scores (0–30), with high scores reflecting poor QoL.

The PSS is the most widely used psychological instrument for measuring the perception of stress. Composed of 10 items rated from “never” to “often”, PSS measures the degree to which situations in one’s life are perceived as stressful [29, 30]. The total score ranges from 10 to 50, and the higher the score, the higher the stress.

The SF12 is a short version of the SF-36, namely a generic instrument to measure population health [31], with a physical composite score and mental composite score calculated based on 12 questions, and the higher the score, the better the physical and the mental quality of life, respectively.

Concurrent validity was established by calculating Pearson correlations between the BoN and the other three validated questionnaires. The data were analyzed using SAS software Version 9.4 (SAS Institute, Cary, NC, USA), with the significance level set at 0.05.

### Test-retest analysis

To assess reproducibility, a test-retest analysis was conducted, with a group of subjects asked to complete the questionnaire twice, with at least a 10-day interval in-between.

### Translation, cross-cultural adaptation, and cognitive debriefing

While the original BoN questionnaire was developed in French, previously-validated methodology was applied to generate an US English-language version, involving cross-cultural validation [32]. The nine steps involved are summarized in Table 1. A number of changes could be implemented throughout the validation process, so as to further improve the initial idiomatic draft.

**Table 1:** Principles of Good Practice for the Translation and Cultural Adaptation Process for Patient-Reported Outcomes (PRO) Measures

Stage	Détails
Preparation	Evaluation of the source text from a linguistic and cultural point of view including definition of concepts
Forward translations	Forward translation into the required target language by two independent translators
Reconciliation	Comparison of the two forward translations to provide the best adaption and produce a draft version of the text
Back translation	Translation of the draft forward translation back into the targeted language without reference to the original language
Back-translation review	Comparison of the original text and the back translation to verify that the meaning of the draft translation is equivalent to source
Analysis and implementation of back-translation review report	Analysis of the back-translation review report to verify if there are changes required to the draft forward
Pilot testing	Clinical review and cognitive debriefing
Review of cognitive debriefing or clinical review results	Review of the results from the cognitive debriefing or clinical review to identify translation modifications necessary for improvement

## Results

### Conceptual phase

Over the preceding 12 months, the psychologist and co-author conducted discussions with 45 NF1 patients on a one-to-one basis concerning their complaints and distresses. Combined with the work of the social worker, this research resulted in the description of the patients’ perceptions in an initial verbatim. Several one-on-one discussions between dermatologist, psychologist, social worker and expert in patient-reported outcomes contributed to consolidate this initial wording, with eventually 17 items forming the conceptual questionnaire.

### Development and validation phase

#### Study population

Patients were selected with the support of the French association of patients with neurofibromatosis, which proposed the questionnaire to previously diagnosed patients. NF1 is a rare skin disease, and working with the Association Neurofibromatoses et Recklinghausen ensured broad recruitment over the territory to guaranteed a reasonably diversity of patients in terms of geographical location, age and sociological status. Overall, 91 patients were contacted of whom 65 responded to the conceptual questionnaire. Among the respondents, 64.6% were women (Table 2).

**Table 2:** Description of the study population

	Men	Women
	*n* = 23 (35.38%)	*n* = 42 (64.62%)
Age	48.87	47.12
Live alone	39.13%	28.57%
Higher level education	56.52%	42.86%
Diagnosis delay, if occurred (years)	10.83	13.28
living in a rural area	30.43%	14.29%
living in a mid size city	47.83%	40.48%
living in a large size city	17.39%	42.86%

The cohort’s mean age was 47.74 years ±17.06 (43.47–52.00). Some 44.4% of patients stated that they were cohabiting, 59.7% exerted a job, and 21.53% were retired, while 47,69% had been to higher level education. Almost all were covered under the French national health insurance, with 78.7% covered under the chronic conditions scheme.

NF1 diagnosis was made by a family physician in 25.9% of cases, a dermatologist in 38.9%, and another specialist in 35.2%. The family physician was involved in managing the disease in 35.4% of cases, and the dermatologist in 75.4%. Altogether, 71.7% of patients stated they were satisfied with their current treatment, and 20% used self-medication.

There was no diagnostic delay for 36.9% of the patients. For those who did experience diagnostic delay, the waiting period was long, since 139.9 months (11.65 years) on average separated the first disease signs from definite diagnosis. About one-third of patients (30.8%) were offered psychological treatment, and 16.9% had actually received it. Overall, 67% of the respondents were in contact with a patients association.

#### Internal validity

Principal component factor exploratory analysis was performed on the 17-item questionnaire. The correlation matrix was previously generated, and the maximum value was 0.8. For all items, neither the answer “never” nor the answer “always” exceeded 20%. Standardized regression coefficients were all > 0.4 (Additional file 1: Table S1), and each group of questions was assigned a dimension, with five dimensions as follows (Table 3):

- Dimension 1, with five questions on concentration and learning problems;
- Dimension 2, with five questions on the way others look at them and the anxiety they feel about the future;
- Dimension 3, with three questions on life with the disease;
- Dimension 4, with two questions on sexuality;
- Dimension 5, with two questions on acceptance and pain.

**Table 3:** Loading of the questions on the factors after rotation

	Factor1	Factor2	Factor3	Factor4	Factor5
Do you think that your concentration problems have had a negative impact on your work?	0.88400	0.00039	0.11713	0.09071	−0.09955
Do you think that your concentration problems have restricted your daily activities?	0.88148	0.30780	0.09345	−0.05582	0.06042
During your education, do you think that you had learning difficulties because of your NF1?	0.79896	−0.00429	0.21201	0.10511	0.06509
Do you think that your concentration problems have hindered your inclusion in society?	0.73315	0.44034	0.26651	−0.03789	0.06598
Have you had any difficulties in asking for help?	0.60144	0.03367	0.57266	0.10930	0.01459
Have you perceived your NF1 as a physical disability?	0.12939	0.88082	0.17472	0.09128	0.01365
Because of your NF1, has the way other people look at you caused you to suffer?	0.05429	0.75423	0.01311	0.31858	0.09864
Has your NF1 affected which clothes you choose to wear?	−0.03823	0.63127	0.34293	0.41378	−0.19180
Have you felt that you have no control over what is happening to you?	0.38068	0.62787	0.33170	−0.05902	0.26411
Are you sometimes afraid of the future because of your NF1?	0.28498	0.55738	0.53923	0.32267	0.09847
Has the paperwork in connection with your NF1 been difficult?	0.16269	0.14724	0.87805	0.06822	0.04185
Have you felt that your socio-economic status may be directly linked to your NF1?	0.31203	0.47696	0.62411	0.02013	0.03600
Have you felt the need to justify yourself?	0.35859	0.31300	0.51316	0.34404	−0.00448
Do you think your NF1 has made you shyer?	−0.14253	0.13130	0.18588	0.81264	0.16481
Has your NF1 hindered your sexuality?	0.30992	0.26387	−0.00112	0.75537	0.04124
Have you succeeded in managing (or dealing with) your NF1 pain?	0.01640	0.11954	0.10323	−0.00897	0.82165
Have you come to terms with the administrative difficulties that you have encountered?	0.01270	−0.0229	−0.0461	0.16370	0.81977

Confirmatory analysis revealed two questions not to be relevant, which were thus removed, resulting in a 15-item questionnaire, with four dimensions:

- Dimension 1, with five questions on concentration and learning problems;
- Dimension 2, with five questions on the way others look at them and the anxiety they feel about the future;
- Dimension 3, with three questions on life with the disease;
- Dimension 4, with two questions on sexuality.

The questionnaire’s uni-dimensionality was confirmed by higher order factor analysis (Additional file 2: Table S2). The practical goodness-of-fit indices were acceptable, with a Bentler comparative fit index of 0.9521 and a Bentler-Bonett non-normed fit index of 0.9355. The model appears well adjusted and well fitted, offering the possibility to group the four dimensions into one single score. All dimensions were found well correlating with the overall score (Table 4).

**Table 4:** Cronbach’s alpha for the four dimensions

	Factor 1	Factor 2	Factor 3	Factor 4
Standardized α	0.90	0.86	0.78	0.62

Cronbach’s alpha coefficient was 0.91 for the entire questionnaire, reflecting its excellent internal coherence, while intradimensional coherences exhibiting good reliability.

#### External validity

The BoN questionnaire highly correlated with the Dermatology Life Quality Index (DLQI), Perceived Stress Scale (PSS), and SF12 mental scores (Table 5). The correlation coefficients between BoN and validated questionnaires were relatively high, confirming BoN’s external validity; the correlation with the SF12 physical score proved the weakest.

**Table 5:** Correlations between the scores

	Pearson correlation coefficients, *N* = 65 Prob > |r| under H0: Rho = 0
DLQI	0.69930 <.0001
PSS	0.72643 <.0001
SF12 -MCS	−0.57650 <.0001
SF12 -Physical	−0.26242 0.0347

*DLQI D*ermatology Life Quality Index*, PSS* Perceived Stress Scale, *PCS* Physical Composite Score /SF12, *MCS* Mental Composite Score /SF12

Cognitive debriefing did not result in any major changes to the questions’ wording.

#### Test-retest analysis

The test-retest reliability was obtained on 23 evaluable subjects (Day 1 and Day 10), demonstrating good reproducibility. The intraclass correlation of each dimension was > 0.85 for each domain.

### Translation and cultural adaptation

The original BoN French version was translated, then linguistically and culturally validated in US English.

While the original BoN questionnaire was developed in French, previously validated methodology was applied in order to generate an US English-language version, involving linguistic and cross-cultural validation 21. This rigorous process comprises a meticulous 9-step procedure and was meant to refine the translation while taking into account nuances of the source document. Briefly, source text evaluation from a linguistic and cultural perspective, including a clear definition of various concepts, was first carried out. In a second step, a separate translation of the text into US English by two independent translators was made. A comparison of the two translations with subsequent text optimization aiming to produce a preliminary draft questionnaire was then performed. Further, a back-translation and back-translation review were performed in which retranslation of the draft questionnaire into the original language and comparison of the original questionnaire against the version obtained from back-translation was done to check whether the overall meaning of the reconciled translation matched that of the source document. Afterwards, to allow pilot testing of the questionnaire, an analysis of the back-translation and implementation of the back-translation review report was performed. Finally, before correction and finalization, a review of cognitive debriefing was performed.

However, from this US English version, an adequate validation in the US patients is still required.

Both versions are presently available (Table 6).

**Table 6:** Burden of adult neurofibromatosis 1 questionnaire in English US and French

Do you think that your concentration problems have had a negative impact on your work?	Pensez-vous que vos difficultés de concentration ont limité vos activités quotidiennes?
Do you think that your concentration problems have restricted your daily activities?	Considérez-vous, que vos difficultés de concentration ont eu un impact négatif dans votre travail?
During your education, do you think that you had learning difficulties because of your NF1?	En raison de votre Neurofibromatose de type 1, lors de votre scolarité, avez-vous rencontré des difficultés d’apprentissage?
Do you think that your concentration problems have hindered your inclusion in society?	Pensez-vous que vos difficultés de concentration ont été un frein à votre intégration dans la société?
Have you had any difficulties in asking for help?	Avez -vous éprouvé des difficultés à demander de l’aide?
Have you perceived your NF1 as a physical disability?	Avez-vous ressenti votre Neurofibromatose de type 1 comme un handicap physique?
Because of your NF1, has the way other people look at you caused you to suffer?	En raison de votre Neurofibromatose de type 1, le regard des autres a-t-il été pénible?
Has your NF1 affected which clothes you choose to wear?	Avez-vous eu le sentiment d’avoir d’un destin imposé?
Have you felt that you have no control over what is happening to you?	Votre Neurofibromatose de type 1, a-t-elle eu une influence sur le choix des vêtements que vous portez?
Are you sometimes afraid of the future because of your NF1?	Vous arrive-t-il, de craindre l’avenir en raison de votre Neurofibromatose de type 1?
Has the paperwork in connection with your NF1 been difficult?	Les démarches administratives en lien avec votre Neurofibromatose de type 1 ont-elles été difficiles?
Have you felt that your socio-economic status may be directly linked to your NF1?	Avez-vous eu le sentiment que votre statut social ait été directement lié à votre Neurofibromatose de type 1?
Have you felt the need to justify yourself?	Avez-vous eu le sentiment d’avoir besoin de vous justifier?
Do you think your NF1 has made you shyer?	Avez-vous pensé que votre Neurofibromatose de type 1 vous rende plus timide?
Has your NF1 hindered your sexuality?	Votre Neurofibromatose de type 1 a-t-elle été un obstacle (un frein) à votre sexualité?
Possible answers to each question and associated score	
“never” or “not applicable” (0)	“jamais” ou non applicable (0)
“rarely” (1)	“rarement” (1)
“sometimes” (2)	“quelques fois “(2)
“often” (3)	“souvent” (3)
“very often” (4)	“très souvent” (4)
“constantly” (5)	“en permanence” (5)

### BoN scoring

The BoN can be expressed as a total score between 0 and 75, where 0 represents no impact and 75 the highest possible impact. The total score is obtained by summing up the scores for each of the 15 questions. In our patient cohort, the mean BoN score obtained was 28.42 (±16.87), the mean score for men being 22.48 (±16.47) versus 31.67 (±16.38) for women (*p* < 0.001). The Shapiro test of normality for the BoN score provided a *p*-value of 0.15, meaning that the hypothesis that the data are normally distributed can-not be rejected.

The respondents evaluated disease severity on a visual analog scale between 1 and 5, with 1 being low severity and 5 very high severity. The subjects were divided into three groups, namely low severity (scores of 1 and 2), moderate severity (3), and high severity (4 and 5). The BoN score of the low severity group was 17.6 (±11.9), while that of the moderate severity group was 29.3 (±14.4), and that of the high severity group 45.5 (±18.2), the BoN score differences statistically significant. Table 7 compares BoN score evolution according to severity, to the evolution of the other validated scores.

**Table 7:** Scores of validated scales according to severity

Severity	N	PSS	DLQI	PCS	MCS	BoN
Low	20	24.34	6.58	52.67	45.63	44.55
Moderate	34	25.23	5.44	46.78	44.04	45.70
High	11	31.82	10.73	47.19	40.08	65.73

*DLQI* Dermatology Life Quality Index, *PSS* Perceived Stress Scale, *PCS* Physical Composite Score /SF12, *MCS* Mental Composite Score /SF12

The mean BoN score was calculated for subjects who stated that they experienced diagnostic delay versus those who did not. BoN score for patients reporting a diagnostic delay was 30.3 (±15.4) versus 23 (±13.9) for those without, the between-group difference being statistically significant (*p* = 0.04).

## Discussion

NF1 has been associated with impaired QoL [33], representing an attack on the patients’ self-esteem and lifestyle [34]. In a cross-sectional study involving 176 adult NF1 cases, participants experienced a significant impact in all aspects of skin-disease-specific QoL [35]. Participants with more visible NF1 signs reported significantly greater overall effects on their skin-disease-specific QoL compared to those with more subtle manifestations. In a Spanish study on NF1 children, an extremely high frequency of cognitive disorders was reported [36]. In an Italian survey involving 129 NF1 adult patients, cosmetic features exerted the greatest impact on QoL [37].

The individual burden is increasingly investigated in the healthcare domain, taking into account QoL, community integration, organization of everyday life, and medical resource consumption [18]. With such specific questionnaires, it is possible to directly evaluate the overarching burden of a particular disease [19, 20, 22, 24]. Due to the advances in QoL research, the pharmaceutical industry, medical device industry, and regulatory agencies are now faced with complex issues related to health-related quality of life claims [38]. Leidy et al. generated recommendations to the healthcare industry for assuring that all health-related QoL claims be based on rigorously-designed studies [38]. Several developments in clinical research have led to a widespread use of questionnaires. The reason for this is the increasing relevance of patient-reported outcome data to achieve market access. Quality of life, patient wellbeing, and patient-centered outcomes are increasingly requested by reimbursement agencies [39].

This report provides support for BoN’s feasibility, reliability, and validity as a specific instrument designed to assess the individual disease burden in adult NF1. The specificity of the BoN questionnaire is that it measures the burden and the impact that NF1 generates in a patient’s daily life. The items are directly derived from discussions conducted by a psychologist with 45 patients with NF1 over a period of 1 year. This is why these items reflect disabilities specific to NF1 such as neurological problems including difficulties in vision, impaired sustained attention, or learning difficulties. Let us add that the burden caused by the disease on many practical aspects of daily activities go beyond the notion of quality of life and are described in the very specific wording expressed by the patients themselves.

With its 15 items and six possible answers for each, this questionnaire is relatively short, understandable and easy-to-use by the patients. BoN has been proven a robust tool, its internal consistency exceeding the minimum reliability criterion of 0.90. This supports using the total BoN score as a distinct measure of the individual disease burden in adult NF1 patients.

In our research, BoN version was able to discriminate between low, moderate, and high disease severity, supporting the tool’s discriminant validity. Consistent with our hypothesis, high BoN scores were associated with adult patients reporting high disease severity on a visual analog scale, whereas low BoN scores were linked to those reporting low disease activity. The mean BoN score proved able to differentiate patients with delayed NF1 diagnosis from those with no delay. Moreover, BoN was shown to exhibit external validity, correlating well with the other validated QoL scales.

Our study exhibits several limitations, the largest one being its relatively small sample size of 65 accessible patients. In this regard, we used a Kaiser-Meyer-Olkin measure to explore the sampling adequacy, and all values were greater than 0.6, except for the 2 items that were removed.

Another limitation is the sole use of BoN in adult patients, as the tool has not yet been validated in children.

We hope that this BoN questionnaire will serve as a valuable tool for healthcare providers to better follow up their patients’ improvement, and adjust NF1 patient management accordingly. This burden evaluation will likely facilitate communication between patients and healthcare providers, improve information transfer, and create a real opportunity for the practitioner to better understand certain issues brought up by the patient.

## Conclusions

The BoN demonstrates feasibility, reliability, and discriminant validity. This instrument can thus be employed to better understand the multidimensional nature of NF1 on the individual burden of adult patients. This tool may similarly have a role to play in the decision-making process.

## Additional files

Additional file 1: Eigenvalues of the factors. (DOCX 15 kb)

Additional file 2: Model assessment parameters. (DOCX 17 kb)

## Abbreviations

BoN: Burden of neurofibromatosis 1
DLQI: Dermatology Life Quality Index
MCS-SF12: Mental Composite Score- Short Form 12
NF1: Neurofibromatosis type 1
PCS-SF12: Physical Composite Score- Short Form 12
PSS: Perceived Stress Scale
QoL: Quality of life
RMSEA: Root mean square error of approximation
