# Supplementary material for: Burden of adult neurofibromatosis 1: development and validation of a burden assessment tool
Source: Orphanet J Rare Dis. 2019 May 3;14:94. doi: 10.1186/s13023-019-1067-8 (PMC6500066; doi:10.1186/s13023-019-1067-8)
Supplement: Supplementary file 1 — Eigenvalues of the factors. (DOCX 15 kb) [file 13023_2019_1067_MOESM1_ESM.docx]

**Additional file 1: Table S1.** Eigenvalues of the factors

|  | **Eigenvalue** | **Difference** | **Proportion** | **Cumulative** |
| --- | --- | --- | --- | --- |
| **1** | 6.83285091 | 4.54561374 | 0.4019 | 0.4019 |
| **2** | 2.28723717 | 0.82942296 | 0.1345 | 0.5365 |
| **3** | 1.45781421 | 0.33368111 | 0.0858 | 0.6222 |
| **4** | 1.12413310 | 0.10702515 | 0.0661 | 0.6884 |
| **5** | 1.01710795 | 0.22071830 | 0.0598 | 0.7482 |
