# Supplementary material for: Burden of adult neurofibromatosis 1: development and validation of a burden assessment tool
Source: Orphanet J Rare Dis. 2019 May 3;14:94. doi: 10.1186/s13023-019-1067-8 (PMC6500066; doi:10.1186/s13023-019-1067-8)
Supplement: Supplementary file 2 — Model assessment parameters. (DOCX 17 kb) [file 13023_2019_1067_MOESM2_ESM.docx]

**Additional file 2: Table S2.** Model assessment parameters

|  | **Summary of goodness of fit tests** | | |
| --- | --- | --- | --- |
|  | **Higher order factor criteria** | required | obtained |
| **Absolute index** | **Ratio of chi-sq to degrees of freedom** | < 5 | 1.36 |
| **Absolute index** | **Pr > chi-sq** | significant | 0.0188 |
| **Absolute index** | **Standardized root mean square residual** | < 0.05 | 0.0913 |
| **Absolute index** | **Goodness of fit index** | > 0.8 | 0.8442 |
| **Parsimonious index** | **Adjusted goodness of fit index** | > 0.8 | 0.7603 |
| **Parsimonious index** | **RMSEA estimate** | Around 0.05 and at the very least under 0.08. The lower the RMSEA, the better the model | 0.0751  CI_90%_: [0.0321–0.1089] |
| **Parsimonious index** | **Akaike Information Criterion** | The lowest value possible of the models tested | 190.1285 |
| **Incremental index** | **Bentler comparative fit index** | > 0.9 | 0.9521 |
| **Incremental index** | **Bentler-Bonett non-normed index** | > 0.9 | 0.9355 |
